# Oesophagopericardial Fistula after Atrial Fibrillation Catheter Ablation

**DOI:** 10.5334/jbsr.1684

**Published:** 2019-01-07

**Authors:** Melissa Grillo, Bruno Coulier, Pierre Deltenre

**Affiliations:** 1 Clinique St Luc, Bouge (Namur), BE

**Keywords:** catheter ablation, complications, atrial fibrillation, fistula, pericardial effusion, oesophageal injury

## Case Report

**Figure 1:**
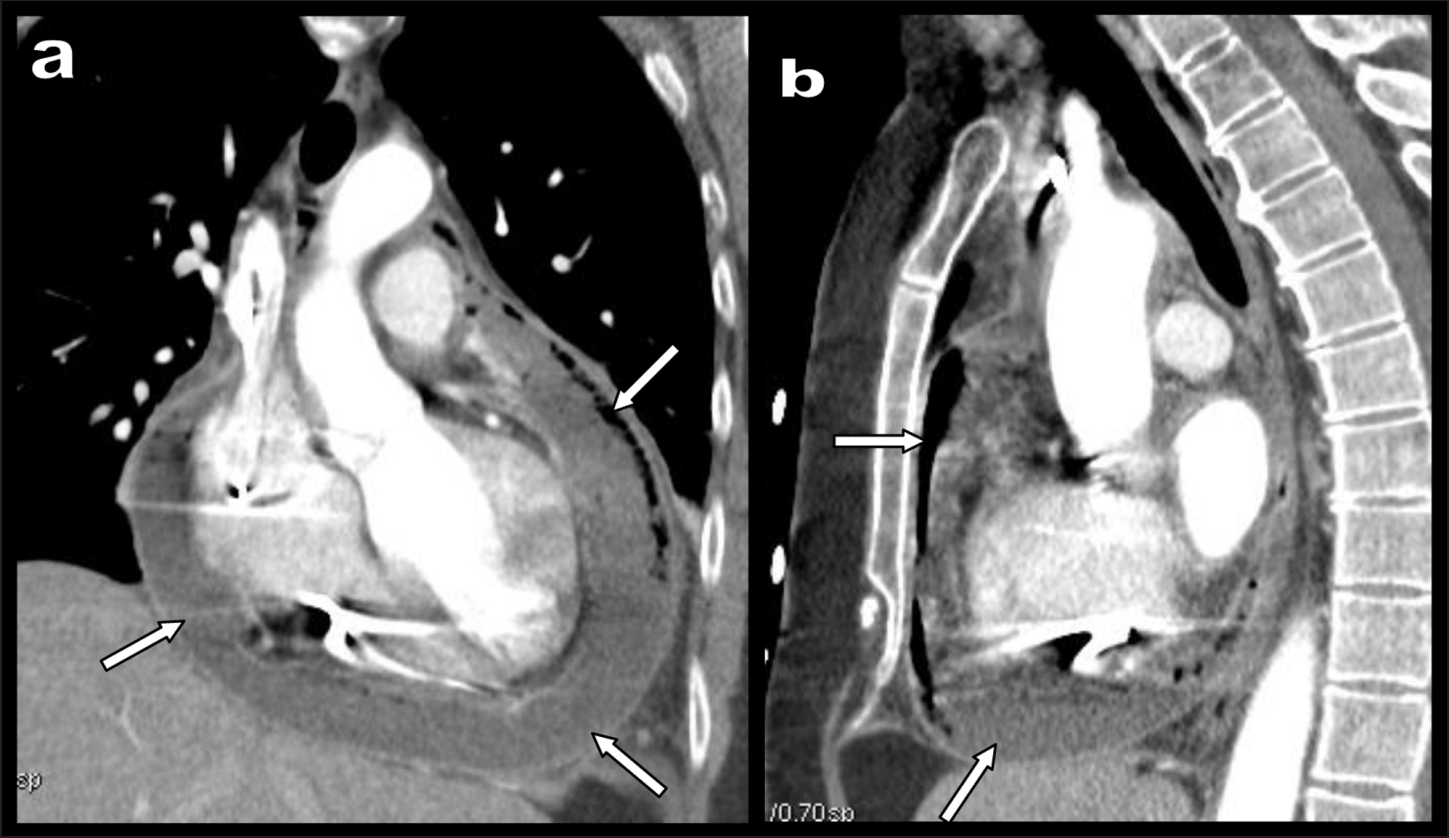


**Figure 2:**
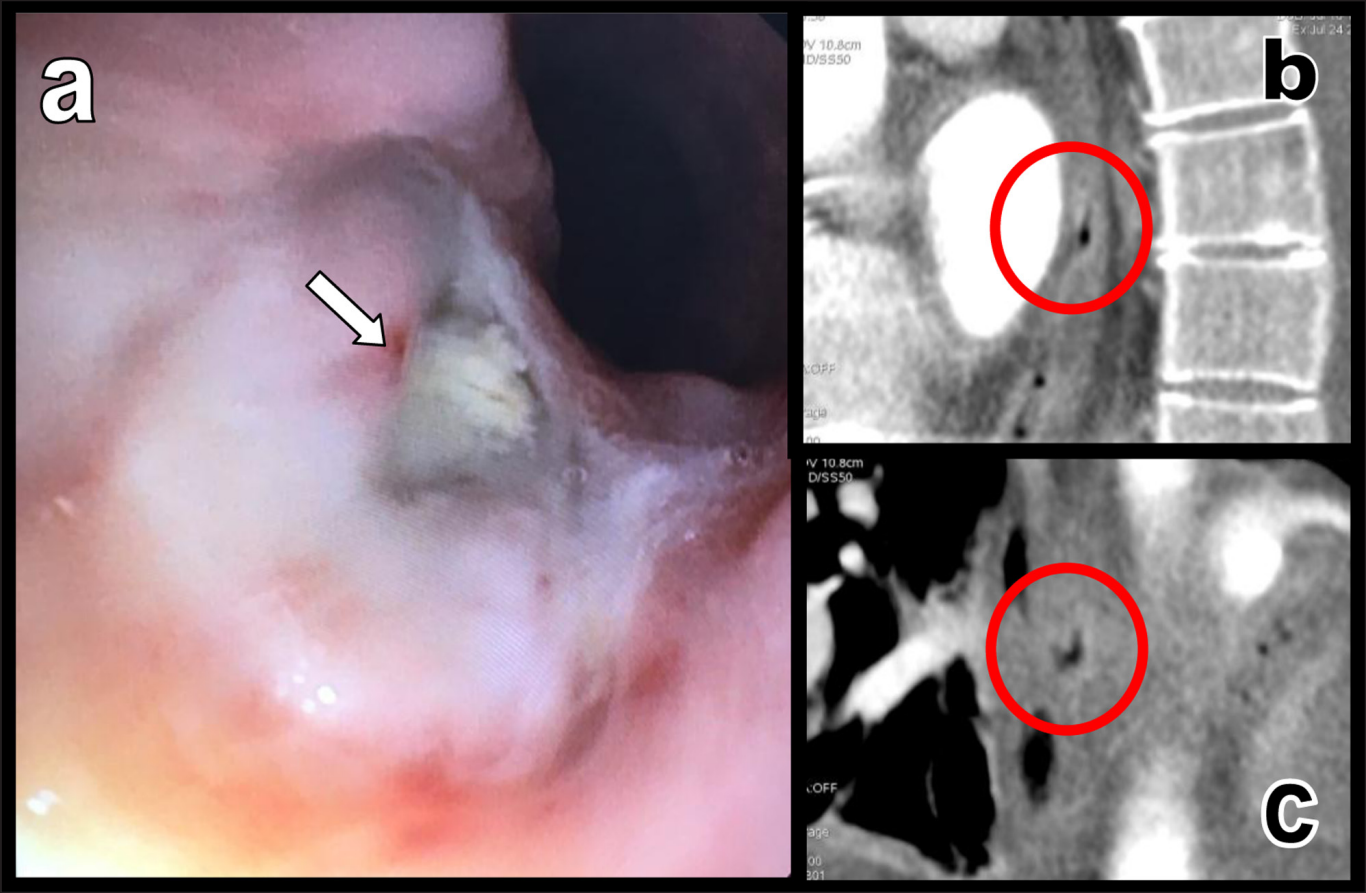


A 54-year-old woman presented with nausea, vomiting, epigastric, and retrosternal crampiform pain three weeks after atrial fibrillation catheter ablation (AFCA). This pain was resistant to analgesics, accentuated by meals, and partially improved by sitting. Biology revealed inflammatory syndrome with CRP at 57 mg/L raising to 441 mg/L in the following 48 hours. Chest computed tomography angiography (CTA) found massive hydropneumopericardium (white arrows on Figure 1) and gastroscopy showed a transparietal ulcer with purulent content on the anterior face of the oesophagus 30 centimetres below the dental arches (white arrow, Figure 2a). Retrospective review of the CT images identified this ulcer just in front of the posterior wall of the left atrial appendage (red circles on Figure 2b and c). Oesophageal-pericardial fistula (OPF) secondary to AFCA was the final diagnosis. Minimally invasive approach associating surgical pericardial drainage and oesophageal stenting to cover the ulcer allowed a slow but effective evolution towards healing.

## Comment

Atrial fibrillation is the most common cardiac arrhythmia, and the success of AFCA is continuously increasing in popularity for patients resistant to medical treatment. The close proximity of the oesophagus with the left atrium places it at risk for injury during AFCA. Various degrees of oesophageal mucosal damage ranging from erythema to deep ulcerations are found in approximately 15% of patients.

Various preventive technical improvements have been implemented to reduce the risk of severe oesophageal injury, but this risk has not been completely eliminated. The thermal insult is believed to start at the oesophageal side to extend in a retrograde way successively into the mediastinum, the pericardium, and then the left atrium.

Atrial-oesophageal fistula (AEF) is the most severe life-threatening complication. It remains fortunately very rare with a reported incidence – potentially underestimated – ranging from 0.04% to 0.2%. It nevertheless presents with an overall mortality rate above 70%. The best management option for AEF remains prompt surgery.

In the rare cases of more limited oesophageal-pericardial fistulas – as reported here – a minimally invasive approach consisting of aggressive antibiotic treatment, pericardial drainage, and oesophageal stenting has been shown effective [1].

Physicians must promptly recognize the signs and symptoms of post-AFCA injury, but the classical delay of onset of symptoms – ranging from two days to five weeks with a peak of occurrence at 10 to 17 days – nevertheless constitutes a diagnostic challenge. OPF is characterized by non-specific thoracic complaints resulting from pericardial effusion and pericarditis accompanied by signs of infection. In AEF additional major complications may be found comprising embolization of air or food components (causing neurological complications or myocardial infarction) and hematemesis and/or haemorrhagic shock caused by bleeding in the oesophagus. OPF can be suspected on CTA by the presence of pericardial effusion with air bubbles in pericardial space. Oesophageal ulceration can be identified. There is often no extravasation of contrast from the left atrium into adjacent spaces.
